# Socio-economic indicators, housing characteristics, site of residence, and substance use as household-level risk factors in GRT-INSPIRED communities

**DOI:** 10.3389/fpubh.2026.1812671

**Published:** 2026-04-13

**Authors:** Tintswalo Mercy Hlungwani, Themba Titus Sigudu, Phoka C. Rathebe, Masilu D. Masekameni, James Wabwire Oguttu

**Affiliations:** 1 Division of Health and Society, School of Public Health, Faculty of Health Sciences, University of the Witwatersrand, Johannesburg, Gauteng, South Africa; 2 Department of Environmental Health, Faculty of Health Sciences, Doornfontein Campus, University of Johannesburg, Johannesburg, Gauteng, South Africa; 3 Department of Development Studies, School of Social Sciences, University of South Africa, Pretoria, South Africa; 4 Department of Agriculture and Animal Health, College of Agriculture and Environmental Sciences, University of South Africa, Johannesburg, Gauteng, South Africa

**Keywords:** household substance use, housing conditions, socio-economic determinants, South Africa, urban health

## Abstract

**Introduction:**

Substance use remains a major public health concern in South Africa and disproportionately affects urban communities experiencing socio-economic disadvantage, poor housing conditions, and spatial inequality. While existing research has largely focused on individual-level substance use, less is known about how household socio-economic characteristics, housing conditions, and place of residence shape household-level substance use in urban settings. This study examined the associations between socio-economic indicators, housing characteristics, site of residence, and household substance use in selected urban communities in Gauteng Province, South Africa.

**Methods:**

A cross-sectional household-level analysis was conducted using secondary data from the GRT-INSPIRED Health and Demographic Surveillance System (HDSS). The study included 46,113 households from three urban sites: Hillbrow, Atteridgeville, and Melusi. Household substance use was defined as the presence of any reported substance used within the household. Socio-economic variables included employment status, income classification, and government grant receipt, while housing variables captured building type, housing disrepair, and the presence of multiple structures. Descriptive analyses were followed by bivariable and multivariable logistic regression to estimate adjusted odds ratios (AORs) and 95% confidence intervals (CIs).

**Results and discussion:**

Alcohol was the most commonly reported substance among households with substance use, reported by 10,453 households (77.1%; 95% CI: 76.4–77.8). In multivariable analysis, households reporting housing disrepair had substantially higher odds of substance use (AOR = 4.43; 95% CI: 3.24–6.05). Residence in Melusi was strongly associated with household substance use compared with Hillbrow (AOR = 4.04; 95% CI: 3.44–4.75). Low-income households had lower odds of substance use (AOR = 0.71; 95% CI: 0.61–0.83), while grant receipt was associated with higher odds (AOR = 1.29; 95% CI: 1.05–1.58). Household substance use in urban Gauteng is strongly shaped by housing quality and spatial context, highlighting the importance of structural and place-based determinants. Integrated urban health interventions addressing housing conditions and neighborhood vulnerabilities are critical for reducing substance-related harm.

## 1 Introduction

Substance use constitutes a major public health concern globally, accounting for a substantial proportion of preventable morbidity, mortality, and social harm (1, 2). Although substance use occurs across all societies, its burden is disproportionately concentrated in low- and middle-income countries (LMICs), where structural inequality, rapid urbanization, and limited access to health and social services amplify vulnerability (3). Increasingly, research has emphasized that substance use is not solely an individual behavior, but is deeply embedded within household, community, and socio-economic contexts. In sub-Saharan Africa, substance use patterns are shaped by a unique combination of historical legacies, demographic transitions, and contemporary socio-economic pressures. Urbanization across the region has accelerated over the past three decades, often outpacing housing provision, employment growth, and social infrastructure (4). As a result, many urban households experience chronic economic insecurity, overcrowding, informal housing arrangements, and high levels of population mobility-conditions that have been associated with psychological distress and increased risk of substance use (5, 6). South Africa presents a particularly salient case. The country has one of the highest levels of income inequality globally, alongside persistently high unemployment, especially among young adults (7). Alcohol remains the most widely consumed psychoactive substance and is a leading contributor to interpersonal violence, road traffic injuries, and non-communicable diseases (8, 9). In parallel, the use of cannabis, opioids (including heroin and *nyaope*), and polysubstance combinations has increased in many urban communities, placing additional strain on households and health systems (10, 11).

Importantly, substance use in South Africa often manifests at the household level, with consequences that extend beyond the individual user. These include financial strain, food insecurity, domestic conflict, child neglect, and reduced social cohesion (12, 13). Yet empirical research has largely focused on individual-level prevalence, leaving household-level dynamics underexplored. Socio-economic disadvantage is widely recognized as a key determinant of substance use, although empirical findings remain context-dependent. Unemployment, unstable income, and limited access to social protection have been linked to increased substance use as a coping response to stress, uncertainty, and social exclusion (14, 15). At the same time, studies in Southern Africa suggest that extreme poverty may constrain access to substances, producing paradoxical patterns in which substance use is more prevalent among households with slightly greater economic resources (16, 17). These competing mechanisms highlight the importance of examining substance use within specific socio-spatial contexts rather than assuming linear relationships. Government social grants play a central role in South Africa's social protection system and reach millions of households. While grants have been shown to reduce poverty and improve food security, their relationship with substance use remains contested, with some studies suggesting protective effects and others indicating potential unintended consequences (18, 19). Understanding how grant receipt intersects with other household vulnerabilities is therefore critical. Housing conditions and neighborhood environments are increasingly recognized as fundamental social determinants of health. International evidence links poor housing quality, overcrowding, and housing insecurity to mental distress, harmful substance use, and reduced capacity for healthy behaviors (20, 21). In South African cities, informal settlements, backyard dwellings, and deteriorating inner-city buildings often coexist with limited access to basic services, exposure to crime, and environmental hazards (22, 23). Place-based effects are particularly pronounced in Gauteng Province, South Africa's economic hub and primary destination for internal and cross-border migrants. High levels of in- and out-migration, combined with stark intra-urban inequalities, create heterogeneous risk environments in which substance use may cluster spatially and socially (24, 25). These dynamics highlight the importance of incorporating site-level and contextual variables into analyses of household substance use.

Urban health and demographic surveillance systems (HDSSs) provide a robust platform for examining the intersection of socio-economic disadvantage, housing conditions, and health outcomes at scale. The GRT-INSPIRED HDSS, operating in diverse urban communities in Gauteng, was explicitly designed to capture not only demographic and health indicators but also contextual and structural determinants through its urban module. This approach aligns with calls for more integrated, systems-oriented analyses of urban health in LMICs (26, 27). Despite growing recognition of the importance of household and contextual determinants, there remains limited empirical evidence on household-level substance use in relation to socio-economic disadvantage and housing conditions in South African urban settings. This study aimed to examine the associations between socio-economic indicators, housing characteristics, site of residence, and household substance use in three urban communities participating in the GRT-INSPIRED HDSS. By focusing on households rather than individuals, the study seeks to generate evidence that is directly relevant for urban policy, social protection, and community-level interventions.

## 2 Materials and methods

### 2.1 Study design and setting

This study employed a cross-sectional household-level analytical design using secondary data generated through the GRT-INSPIRED (Gauteng Research Triangle–Inspired Spatial Planning, Infrastructure Development and Employment) Health and Demographic Surveillance System (HDSS). The HDSS operates in three urban communities in Gauteng Province, Hillbrow, Atteridgeville, and Melusi, selected to reflect diverse socio-demographic, spatial, and housing contexts. Hillbrow is a high-density inner-city area characterized by substantial population mobility and a high proportion of residents from multiple nationalities. Atteridgeville represents a long-established township setting with large, multi-generational household structures, while Melusi is a predominantly young, rapidly growing informal settlement. These distinct settings required adaptable and context-sensitive fieldwork approaches and informed the inclusion of site as a key analytical variable.

### 2.2 Study population

The study population comprised 46 113 households enumerated through routine GRT-INSPIRED data collection activities. All households with available data on household substance use and core socio-economic and housing variables were eligible for inclusion. Households with missing outcome data were excluded from inferential analyses. Missing values in exposure variables were retained and reported descriptively to reflect real-world data completeness challenges in highly mobile urban populations.

### 2.3 Data collection

Household data were collected by trained fieldworkers using electronic data capture devices, enabling the collection of informed consent, household rosters, and detailed questionnaire responses aligned with the SAPRIN core protocol and the GRT-INSPIRED urban module. Fieldworkers operated across all three sites and engaged with households under diverse and often challenging conditions, including variable weather and high-density living environments. Fieldworkers received continuous training throughout the year, with refresher sessions conducted weekly or as required in response to emerging field or data quality issues. The fieldwork model emphasized both technical competence and interpersonal engagement, recognizing that effective interaction with respondents was essential for accurate data capture across culturally and socially heterogeneous communities.

### 2.4 Data quality assurance and management

Data quality was ensured through a multi-layered validation and correction process. Following the adoption of the SAPRIN Util system, data were ingested nightly and subjected to automated checks for completeness, consistency, and logical validity. Records that failed validation were flagged, and fieldworkers returned to households the following day to correct errors or complete missing information. Initial data checks and corrections were conducted at site offices each evening. Data were then synchronized to the central data center based at the School of Public Health, University of the Witwatersrand, where Survey Solutions software was used for further collation and verification. A dedicated data management team conducted ongoing data cleaning, including script-based error detection, validation checks, and reconciliation procedures. Given the high levels of population mobility in Gauteng Province, particular attention was paid to migration reconciliation. This process involved distinguishing between internal migration within surveillance nodes and external migration out of the HDSS area, with follow-up conducted to determine whether movements were temporary or permanent. Accurate measurement of in- and out-migration is a core component of the HDSS and essential for the validity of longitudinal and cross-sectional analyses.

### 2.5 Outcome variables

The primary outcome was household substance use, defined as the presence of at least one reported substance used within the household at the time of assessment. Household substance use was operationalized as a binary household-level indicator denoting whether at least one household member was reported to use any substance at the time of assessment. This measure therefore captures the presence of substance use within the household, rather than the number of users, frequency of use, or severity of use. Substance use was analyzed as a binary variable (yes/no). Among households reporting substance use, descriptive analyses were undertaken to characterize the distribution of specific substances, including alcohol, cannabis (dagga), and other licit and illicit substances.

### 2.6 Exposure variables

Socio-economic exposure variables were derived from household-level classifications recorded in the GRT-INSPIRED HDSS dataset. Household employment status reflected the household-level employment classification recorded in the surveillance data. This variable represents the overall employment status assigned to the household based on the HDSS questionnaire and does not necessarily indicate that all household members are unemployed or employed. Rather, it serves as a proxy measure of the household's socio-economic position. Household income classification reflected the HDSS categorization of household income status, derived from self-reported household income and related socio-economic indicators collected during routine surveillance. Households were categorized into relative income groups (e.g., low, middle, and high income) based on predefined thresholds within the HDSS framework, reflecting socio-economic position within the study population rather than exact income amounts.

### 2.7 Statistical analysis

All analyses were conducted using STATA software version 19. Descriptive statistics were used to summarize socio-economic characteristics, housing conditions, and substance use patterns, reported as frequencies, percentages, and 95% confidence intervals (CIs). Bivariable associations between household substance use and socio-economic, housing, and site variables were assessed using χ^2^ tests, with statistical significance set at p < 0.05. Variables demonstrating significant bivariable associations, as well as those identified a priori based on theoretical relevance, were entered into a multivariable logistic regression model to estimate adjusted odds ratios (AORs) and 95% CIs. The final model included site of residence, employment status, income classification, grant receipt, housing disrepair, and the presence of multiple structures on the dwelling. Results were interpreted with consideration of potential confounding and collinearity.

### 2.8 Ethical considerations

Ethical approval for this study was obtained from the Human Research Ethics Committee (Medical) of the University of the Witwatersrand (ETHICS REFERENCE NO: 200908, date of approval: 04 June 2025). The analysis used secondary, de-identified household-level data collected through the GRT-INSPIRED Health and Demographic Surveillance System (HDSS). Formal permission to conduct the study and access the dataset was obtained from the GRT-INSPIRED HDSS through a signed data use agreement with the SAPRIN GRT-Inspired node. All primary data collection within the HDSS was conducted in accordance with the SAPRIN core protocol, with informed consent obtained from participating households prior to data collection. For the present study, only anonymized data were accessed, and no personally identifiable information was available to the research team. The secondary analysis complied with all relevant ethical guidelines for research involving human participants. Data were handled with strict confidentiality, stored in secure, password-protected environments, and used solely for academic and research purposes. No direct contact with participants was required for this study.

## 3 Results

### 3.1 Descriptive statistics

#### 3.1.1 Socio-economic characteristics of households by site

As shown in Table 1, the socio-economic characteristics of the 46 113 households included in the study indicate a population with notable labor market vulnerability and broadly comparable socio-economic profiles across the three surveillance sites of Hillbrow, Atteridgeville, and Melusi.

**Table 1:** Socio-economic characteristics of households by site of residence.

Variable	Category	Total participants			Hillbrow			Atteridgeville			Melusi		
		*n*	%	95% CI	*n*	%	95% CI	*n*	%	95% CI	*n*	%	95% CI
Employment status	Employed	17 157	37.2	36.8–37.6	9 483	37.2	36.6–37.8	4 637	37.2	36.3–38.0	3 037	37.2	36.2–38.3
	Unemployed	18 072	39.2	38.7–39.6	9 953	39.0	38.4–39.6	4 883	39.2	38.3–40.1	3 236	39.7	38.6–40.7
	Unknown	10 884	23.6	23.2–24.0	6 052	23.7	23.2–24.3	2 949	23.6	22.9–24.4	1 882	23.1	22.2–24.0
Household income classification	Low income	16 675	36.2	35.7–36.6	9 217	36.2	35.6–36.8	4 503	36.1	35.3–37.0	2 955	36.2	35.2–37.2
	Middle income	13 755	29.8	29.4–30.2	7 605	29.8	29.3–30.4	3 713	29.8	29.0–30.6	2 437	29.9	28.9–30.9
	High income	2 114	4.6	4.4–4.8	1 170	4.6	4.3–4.8	571	4.6	4.2–4.9	373	4.6	4.1–5.0
	Unknown	13 569	29.4	29.0–29.8	7 496	29.4	28.8–30.0	3 682	29.5	28.7–30.3	2 391	29.3	28.3–30.3
Recipient of government grant	Yes	8 935	19.3	19.0–19.7	4 938	19.4	18.9–19.9	2 412	19.3	18.6–20.0	1 585	19.4	18.6–20.3
	No	37 178	80.6	80.3–81.0	20 550	80.6	80.1–81.1	10 057	80.7	80.0–81.4	6 570	80.6	79.7–81.4

*n*, Frequency or count; %, Percentage; 95% CI, 95% Confidence interval.

Overall, 37.2% (*n* = 17 157; 95% CI: 36.8–37.6) of households reported being employed, while a slightly higher proportion, 39.2% (*n* = 18 072; 95% CI: 38.7–39.6), were unemployed. In addition, 23.6% (*n* = 10 884; 95% CI: 23.2–24.0) of households had unknown employment status, reflecting incomplete reporting in the surveillance dataset. The distribution of employment status was remarkably consistent across the three sites. In Hillbrow, 37.2% (*n* = 9 483; 95% CI: 36.6–37.8) of households were employed and 39.0% (*n* = 9 953; 95% CI: 38.4–39.6) were unemployed, while 23.7% (*n* = 6 052; 95% CI: 23.2–24.3) had unknown status. Similarly, in Atteridgeville, 37.2% (*n* = 4 637; 95% CI: 36.3–38.0) of households were employed and 39.2% (*n* = 4 883; 95% CI: 38.3–40.1) were unemployed, with 23.6% (*n* = 2 949; 95% CI: 22.9–24.4) classified as unknown. In Melusi, 37.2% (*n* = 3 037; 95% CI: 36.2–38.3) of households reported employment and 39.7% (*n* = 3 236; 95% CI: 38.6–40.7) unemployment, while 23.1% (*n* = 1 882; 95% CI: 22.2–24.0) had unknown employment status.

Household income classification similarly reflects the socio-economic context of the study population. Overall, 36.2% (*n* = 16 675; 95% CI: 35.7–36.6) of households were classified as low income, while 29.8% (*n* = 13 755; 95% CI: 29.4–30.2) fell within the middle-income category. Only 4.6% (*n* = 2 114; 95% CI: 4.4–4.8) were classified as high-income households, indicating that economically advantaged households constituted a small minority of the study population. A further 29.4% (*n* = 13 569; 95% CI: 29.0–29.8) had unknown income classification, which may reflect challenges in collecting income information in complex urban household contexts. Notably, the distribution of income categories was highly similar across the three sites. Low-income households accounted for 36.2% (*n* = 9 217) in Hillbrow, 36.1% (*n* = 4 503) in Atteridgeville, and 36.2% (*n* = 2 955) in Melusi. Middle-income households comprised 29.8% (*n* = 7 605) in Hillbrow, 29.8% (*n* = 3 713) in Atteridgeville, and 29.9% (*n* = 2 437) in Melusi. High-income households represented 4.6% of households in each site.

With respect to social protection, 19.3% (*n* = 8 935; 95% CI: 19.0–19.7) of households reported receiving a government grant, while the majority 80.6% (*n* = 37 178; 95% CI: 80.3–81.0) did not receive any grant support. The prevalence of grant receipt was again very similar across sites. In Hillbrow, 19.4% (*n* = 4 938; 95% CI: 18.9–19.9) of households reported receiving a grant; in Atteridgeville, the proportion was 19.3% (*n* = 2 412; 95% CI: 18.6–20.0); and in Melusi, 19.4% (*n* = 1 585; 95% CI: 18.6–20.3) of households reported grant receipt.

#### 3.1.2 Housing characteristics and dwelling conditions

Table 2 presents the distribution of housing characteristics and dwelling conditions among the 46 113 households included in the study. Just over half of households resided in multi-floor buildings (55.2%; 95% CI: 54.8–55.7), while the remaining 44.8% (95% CI: 44.3–45.2) occupied single-floor structures.

**Table 2:** Housing characteristics and dwelling conditions.

Variable	Category	*n* ^a^	% ^b^	95% CI ^c^
Building type	Multi-floor	25 475	55.2	54.8–55.7
	Single-floor	20 638	44.8	44.3–45.2
Building structures on dwelling	≥2 structures	15 289	33.2	32.8–33.6
	Single structure	30 824	66.8	66.4–67.2
Dwelling functional status	Functional	45 011	97.6	97.5–97.7
	Not functional	1 102	2.4	2.2–2.5
Repairs needed (fix damaged parts)	Yes	2 405	5.2	5.0–5.4
	No	43 708	94.8	94.6–95.0

*n*, Frequency or count; %, Percentage; 95% CI, 95% Confidence interval.

Regarding the number of structures per dwelling, approximately one-third of households reported the presence of two or more structures on the same plot (33.2%; 95% CI: 32.8–33.6), whereas two-thirds occupied a single structure (66.8%; 95% CI: 66.4–67.2). Most dwellings were reported to be functional at the time of the survey (97.6%; 95% CI: 97.5–97.7), with only a small proportion classified as not functional (2.4%; 95% CI: 2.2–2.5). Similarly, the need for repairs was uncommon, with 5.2% (95% CI: 5.0–5.4) of households indicating damaged components requiring repair, compared with 94.8% (95% CI: 94.6–95.0) reporting no need for repairs.

#### 3.1.3 Distribution of substances among households in the study population

As shown in Table 3, substance use was reported in 13 555 households, representing 29.4% (95% CI: 29.0–29.8) of the 46 113 households included in the study. This indicates that nearly one in three households reported the presence of at least one substance used by a household member.

**Table 3:** Distribution of substances among households in the study population.

Substance	Households (*n*)	Households (%)	95% CI
Any substance use	13 555	29.4	29.0–29.8
Alcohol	10 453	22.7	22.3–23.1
Cannabis (dagga)	1 484	3.2	3.0–3.4
Other substances	3 775	8.2	7.9–8.4
Nyaope/heroin/whoonga	103	0.2	0.2–0.3
Sleeping tablets	75	0.2	0.1–0.2
Glue/petrol/thinners (sniffing)	16	0.03	0.01–0.05
Cocaine/crack	15	0.03	0.01–0.05

Among the substances reported, alcohol was by far the most prevalent, reported by 10 453 households, corresponding to 22.7% (95% CI: 22.3–23.1) of all households in the study population. This finding highlights the dominant role of alcohol within household-level substance use patterns in the surveyed urban communities.

Cannabis (dagga) was the second most commonly reported specific substance, although at substantially lower levels than alcohol, being reported in 1 484 households (3.2%; 95% CI: 3.0–3.4). In addition, other substances, which include substances not separately categorized in the table, were reported by 3 775 households (8.2%; 95% CI: 7.9–8.4), indicating that a notable minority of households reported exposure to substances beyond alcohol and cannabis.

The prevalence of high-risk illicit substances was relatively low. Nyaope, heroin, or whoonga were reported in 103 households (0.2%; 95% CI: 0.2–0.3), while the use of sleeping tablets was reported in 75 households (0.2%; 95% CI: 0.1–0.2). The use of inhalants such as glue, petrol, or thinners was rare, reported in only 16 households (0.03%; 95% CI: 0.01–0.05). Similarly, cocaine or crack use was reported in 15 households (0.03%; 95% CI: 0.01–0.05).

#### 3.1.4 Inferential statistics

##### 3.1.4.1 Bivariable association between socio-economic indicators and household substance use

As shown in Table 4, several socio-economic and housing characteristics were significantly associated with household substance use in the bivariable analysis. With respect to employment status, households classified as employed had the highest prevalence of substance use, with 34.7% (*n* = 5 954) reporting substance use. In contrast, unemployed households showed a lower prevalence of 23.8% (*n* = 4 303), while households with unknown employment status reported a prevalence of 30.3% (*n* = 3 298). The association between employment status and household substance use was statistically significant (*p* < 0.001), suggesting that labor market participation may influence patterns of substance exposure within households.

**Table 4:** Bivariable association between socio-economic indicators and household substance use.

Variable	Category	Total households (*n*)	Households with substance use		*p*-value
			*n*	%	
Employment status	Employed	17 157	5 954	34.7	< 0.001
	Unemployed	18 072	4 303	23.8	
	Unknown	10 884	3 298	30.3	
Household income classification	Low income	16 675	4 727	28.3	< 0.001
	Middle/High income	15 869	5 527	34.8	
	Unknown	13 569	3 301	24.3	
Recipient of government grant	Yes	8 935	2 299	25.7	< 0.001
	No	37 178	11 248	30.2	
Housing disrepair (repairs needed)	Yes	2 405	1 109	46.1	< 0.001
	No	43 708	12 446	28.5	
Multiple structures on dwelling	Yes	15 289	389	2.5	0.025
	No	30 824	2 284	7.4	
Dwelling functional status	Not functional	1 102	192	17.4	< 0.001
	Functional	45 011	13 363	29.7	
Site	Hillbrow	25 488	7 468	29.3	< 0.001
	Atteridgeville	12 469	3 721	29.8	
	Melusi	8 155	2 366	29.0	

Household income classification also demonstrated a significant association with substance use (*p* < 0.001). Households in the middle- or high-income categories had the highest prevalence of substance use at 34.8% (*n* = 5 527), compared with 28.3% (*n* = 4 727) among low-income households. Households with unknown income classification had the lowest reported prevalence of substance use at 24.3% (*n* = 3 301). These findings suggest a complex relationship between socio-economic status and household substance use, where substance use does not necessarily increase with greater economic deprivation.

Similarly, government grant receipt was significantly associated with household substance use (*p* < 0.001). Households not receiving grants had a higher prevalence of substance use (30.2%; *n* = 11 248) compared with 25.7% (*n* = 2 299) among grant-recipient households. Housing conditions showed particularly strong associations with household substance use. Households reporting housing disrepair, defined as the need for repairs, had the highest prevalence of substance use at 46.1% (*n* = 1 109), compared with 28.5% (*n* = 12 446) among households that did not report repair needs (*p* < 0.001). This finding suggests that poor housing conditions may be an important contextual determinant of substance exposure at the household level.

The presence of multiple structures on a dwelling was also associated with substance use (p = 0.025), although the pattern was less pronounced. Households with multiple structures reported a prevalence of 2.5% (*n* = 389) compared with 7.4% (*n* = 2 284) among households with a single structure.

### 3.2 Multivariable analysis of socio-economic factors associated with household substance use

Table 5 presents the results of the multivariable logistic regression analysis examining socio-economic and housing-related factors independently associated with household substance use. After adjustment for all variables included in the model, both site of residence and household-level characteristics remained important predictors. Site of residence showed a strong and statistically significant association with household substance use. Households located in Melusi had more than four times higher odds of reporting substance use compared with those in Hillbrow (AOR = 4.04; 95% CI: 3.44–4.75; *p* < 0.001), highlighting substantial geographic disparities that persist even after controlling for socio-economic and housing conditions.

**Table 5:** Multivariable logistic regression of socio-economic factors associated with household substance use.

Variable	AOR ^a^	95% CI ^b^	*p*-value
Site (Melusi vs. Hillbrow)	4.04	3.44–4.75	< 0.001
Unemployed	0.86	0.73–1.01	0.068
Low income	0.71	0.61–0.83	< 0.001
Grant recipient	1.29	1.05–1.58	0.016
Repairs needed (housing disrepair)	4.43	3.24–6.05	< 0.001
Multiple structures on dwelling	1.13	0.92–1.37	0.239

AOR, Adjusted Odds Ratio; 95% CI, 95% Confidence interval.

Housing-related factors emerged as some of the strongest independent predictors. Households reporting the need for repairs, an indicator of housing disrepair, had markedly higher odds of substance use (AOR = 4.43; 95% CI: 3.24–6.05; *p* < 0.001). In contrast, the presence of multiple structures on a dwelling was not independently associated with substance use after adjustment (AOR = 1.13; 95% CI: 0.92–1.37; *p* = 0.239), suggesting that its effect in the bivariable analysis was likely confounded by other housing or site-level factors. Socio-economic indicators showed mixed associations. Low-income households had significantly lower odds of substance use compared with higher-income households (AOR = 0.71; 95% CI: 0.61–0.83; *p* < 0.001), while receipt of a government grant was associated with increased odds of substance use (AOR = 1.29; 95% CI: 1.05–1.58; *p* = 0.016). Unemployment was not independently associated with household substance use after adjustment, although the estimate suggested a borderline protective effect (AOR = 0.86; 95% CI: 0.73–1.01; *p* = 0.068).

## 4 Discussion

This study examined the associations between socio-economic indicators, housing characteristics, and site of residence with household-level substance use across three urban communities in Gauteng Province. Using data from over 46 000 households, the findings provide important insights into how structural and contextual factors shape household substance exposure in rapidly urbanizing South African settings.

Overall, substance use was reported in 29.4% of households, indicating that nearly one in three households had at least one member using a substance. Alcohol accounted for the largest share of substance exposure, being reported in 22.7% of households, substantially exceeding the prevalence of other substances. Cannabis was the second most commonly reported substance, although at considerably lower levels. These findings are consistent with national and regional evidence showing that alcohol remains the most widely consumed psychoactive substance in South Africa and a major contributor to preventable health and social harms (8, 10). The relatively low prevalence of high-risk illicit substances such as nyaope, heroin, inhalants, and cocaine suggests that household substance exposure in the study communities is primarily driven by alcohol consumption rather than by illicit drug markets. However, even relatively low prevalence of these substances may still have important health and social implications given their strong association with addiction, crime, and community instability (10, 11).

The socio-economic profile of the study population reflected substantial labor market vulnerability. Across all three surveillance sites, unemployment slightly exceeded employment, and a considerable proportion of households had missing employment or income information. These findings are consistent with broader evidence describing persistent unemployment and labor market precarity in South African urban economies (7, 28). Despite these structural constraints, the distribution of employment status, income classification, and grant receipt was remarkably similar across Hillbrow, Atteridgeville, and Melusi. This suggests that the three sites share broadly comparable socio-economic conditions despite their different urban forms, ranging from inner-city high-density housing to township and informal settlement contexts.

Interestingly, the bivariable analysis demonstrated that households classified as employed or belonging to middle- or higher-income categories reported a higher prevalence of substance use compared with low-income households. This pattern challenges the assumption that substance use increases monotonically with economic deprivation. Instead, the findings support emerging evidence suggesting a non-linear relationship between socio-economic status and substance use in low- and middle-income contexts, where extremely poor households may have limited access to substances due to affordability constraints (29, 30). In urban environments characterized by inequality and informal economic activity, households with slightly greater financial resources may have increased access to alcohol and other substances through social networks, retail outlets, and informal markets.

The relationship between government grant receipt and household substance use also warrants careful interpretation. In the bivariable analysis, households not receiving grants had a slightly higher prevalence of substance use compared with grant recipients. However, after adjustment in the multivariable model, grant receipt was associated with increased odds of household substance use. This finding should not be interpreted as evidence that social grants promote substance use. Instead, grant receipt likely serves as a marker of underlying household vulnerability, including chronic poverty, caregiving responsibilities, disability, or long-term unemployment. Previous research has demonstrated that social grants play an important role in reducing poverty, improving food security, and supporting household stability in South Africa (31, 32). At the same time, households experiencing structural stressors may still face psychosocial challenges that contribute to substance use behaviors (33). The interaction between social protection, stress, and community-level substance availability therefore requires further investigation.

Housing conditions emerged as one of the strongest predictors of household substance use. Households reporting the need for repairs had a substantially higher prevalence of substance use in the bivariable analysis and remained strongly associated with substance use in the adjusted model. Specifically, housing disrepair was associated with more than fourfold higher odds of substance use after controlling for socio-economic and contextual factors. These findings reinforce the growing body of literature recognizing housing quality as a critical social determinant of health (20, 34). Poor housing conditions may contribute to chronic stress, reduced psychological wellbeing, and weakened social cohesion, all of which have been linked to increased reliance on alcohol or other substances as coping mechanisms (34, 35). In dense urban environments where housing infrastructure is deteriorating or poorly maintained, such stressors may accumulate and amplify health risk behaviors.

Spatial context also played an important role in shaping household substance use patterns. Although the descriptive distribution of substance use across the three sites appeared similar, the multivariable analysis revealed that households located in Melusi had more than four times higher odds of reporting substance use compared with those in Hillbrow. This finding highlights the importance of place-based determinants of health. Informal settlements and rapidly growing peri-urban communities often face limited infrastructure, overcrowding, weak regulatory environments, and high exposure to social stressors. These conditions can create environments where substance use behaviors become normalized or more accessible (36, 37). The persistence of site-level effects even after adjustment for socio-economic and housing characteristics suggests that neighborhood dynamics—such as alcohol outlet density, social norms, crime exposure, and community cohesion—may play a critical role in shaping substance use patterns (38).

Several limitations should be considered when interpreting these findings. First, household substance use was measured as a binary indicator reflecting the presence of at least one household member using a substance, and therefore does not capture the frequency, intensity, or number of substance-using individuals within the household. As a result, households with occasional substance use are analytically grouped with households where multiple members may engage in frequent or harmful use. Second, the cross-sectional design precludes causal inference, making it difficult to determine whether socio-economic or housing conditions lead to substance use, or whether substance use contributes to deteriorating household conditions. Third, some socio-economic variables were available only as household-level categorical proxies and may not fully capture intra-household economic dynamics or employment participation.

Despite these limitations, the study has several strengths. It draws on a large, population-based dataset covering more than 46 000 households across diverse urban contexts, allowing for robust statistical analysis and comparison across sites. The integration of socio-economic, housing, and spatial variables provides a more comprehensive understanding of household substance use than studies focusing solely on individual-level determinants.

Overall, the findings underscore that household substance use in urban South Africa is shaped by a complex interaction of socio-economic conditions, housing quality, and neighborhood environments. Addressing substance-related harms in such contexts will therefore require integrated urban health strategies that extend beyond individual behavioral interventions to include improvements in housing infrastructure, social protection systems, and community-level environments.

## Data Availability

The original contributions presented in the study are included in the article/supplementary material, further inquiries can be directed to the corresponding author.
